# Advocating for children's surgery within country health plans: lessons from Nigeria and the global stage

**DOI:** 10.3389/fpubh.2023.1209902

**Published:** 2023-08-08

**Authors:** Justina O. Seyi-Olajide, Omolara Faboya, Omolara Williams, Kokila Lakhoo, Emmanuel A. Ameh

**Affiliations:** ^1^Department of Surgery, Lagos University Teaching Hospital, Idi-Araba, Lagos, Nigeria; ^2^Department of Surgery, Lagos State University Teaching Hospital, Ikeja, Lagos, Nigeria; ^3^Department of Surgery, Lagos State University College of Medicine, Ikeja, Lagos, Nigeria; ^4^Department of Surgery, University of Oxford, Oxford University Hospitals, Oxford, United Kingdom; ^5^Department of Surgery, National Hospital, Abuja, Nigeria

**Keywords:** children's surgery, national surgical plan, national health plan, advocacy, Nigeria

## Abstract

**Background:**

Despite the growing emphasis on provision of quality safe and affordable surgical care in low- and middle-income countries, and the World Health Assembly resolution 68. 15 on strengthening emergency and essential surgical care and anesthesia as components of universal health coverage, a review of published surgical plans of various countries, revealed a lack of emphasis on children's surgery. Due to the peculiarities of the human resource, infrastructure and equipment required for children's surgery, a lack of deliberate actions and policies targeted at strengthening surgical care for children implies that achieving universal health coverage for children may not be a reality in this setting.

**Methods:**

A baseline assessment of children's surgical capacity was conducted in Nigeria as a part of the National Surgical Obstetrics Anesthesia and Nursing Plan (NSOANP) process. The assessment was done using the World Health Organization (WHO) hospital assessment tool modified for children's surgery (Children Surgical Assessment Tool).

**Results:**

Significant infrastructural gaps were found, with an abysmally low density of pediatric surgeons and anaesthesiologists, poor emergency preparedness, lack of reliable surgical data and non-inclusion of children's surgery in the national strategic health plan. Using the Global Initiative for Children's Surgery's (GICS) Optimal Resources for Children's Surgical Care (OReCS) document and focusing on the strategic goals and priorities, children's surgery was incorporated into the NSOANP. Implementation of the plan is currently ongoing.

**Conclusion:**

From Nigeria's experience, appropriate advocacy and inclusion of children surgery providers in policy making will promote prioritization of children's surgery in country health and surgical plans.

## Introduction

In recent years, there have been increasing emphasis on scaling-up access to quality safe and affordable surgical care in low- and middle-income countries where most of the estimated 1.7 billion children and adolescents without access to surgical care live (1). Within the region, about 85% of children will develop a potentially treatable surgical condition before the age of 15 years (2). The world health assembly (WHA) resolution 68.15 which mandated countries to strengthen emergency and essential surgical care and anesthesia as a component of universal health coverage culminated in countries developing surgical plans (3). These plans have served as roadmaps to achieve the goal of the resolution. While a few countries have launched national surgical plans, even fewer have commenced implementation of their plans. A review of these plans reveals a significant lack of specific focus on children's surgery. This scenario puts children's surgery at risk of being excluded from policies targeted at strengthening surgical care, advocacy and investment. Children require age and size appropriate equipment, children-specific infrastructure and specialized personnel for safe and quality surgical care. Without deliberate actions to strengthen children's surgery, achieving universal health coverage in children may not be a reality.

The need for a national surgical plan in Nigeria, presented an excellent opportunity to advocate for children's surgery to policy makers, development partners and other stakeholders.

The aim of this report is to highlight the lessons from Nigeria and the global children surgery space to support the inclusion of children's surgery within country health plans.

## Materials and methods

Nigeria has an estimated population of nearly 217 million of which about 43% are children aged between 0 and 14 years (4, 5). The country is divided into 6 geopolitical zones which are distinct in culture, resources and health care capacity. Nigeria's current National Strategic Health Development Plan II did not prioritize surgical care. In the same document, the section on evaluation of the state of child and adolescent healthcare laid no emphasis on surgery (6).

### Baseline assessment of children's surgical capacity in Nigeria

**Figure 1:**
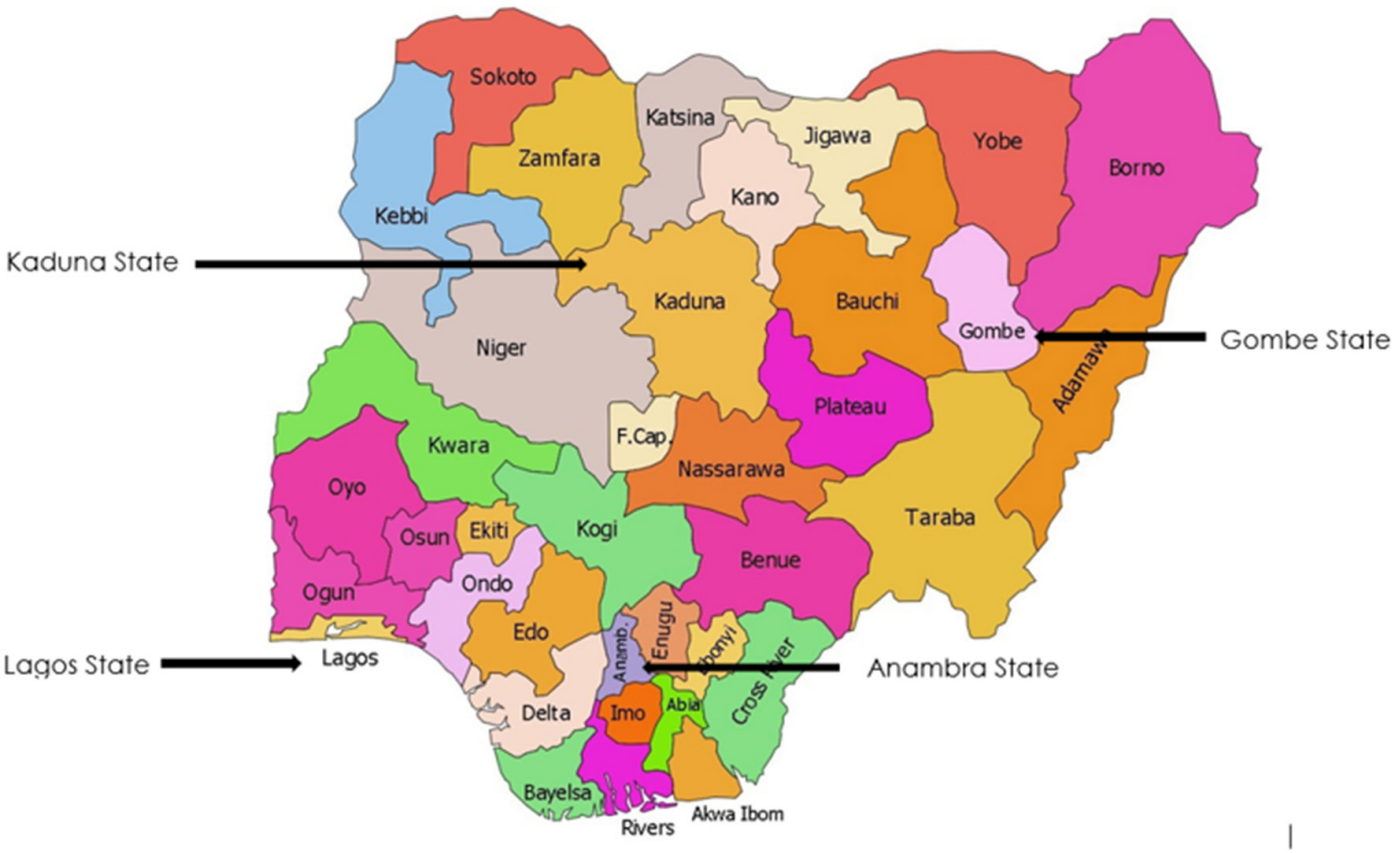
Four states included in the baseline assessment (Map obtained from https://paintmaps.com/blank-maps/301/samples).

To define the status of children's surgery in Nigeria, a baseline assessment was conducted as part of the National Surgical Obstetrics Anesthesia and Nursing Plan (NSOANP) process. A convenient sample of 28 district hospitals (based on accessibility and available resources for evaluation) in 4 states (1 from each of 4 geopolitical zones) were evaluated for their capacity to provide safe, affordable and quality children's surgical care (Figure 1, map of Nigeria with the spread). The total number of public and faith-based secondary level (district level) hospitals was 93 out of which 25 (26.9%) were accessed. In addition, 3 out of the 7 tertiary level hospitals in the same states were also assessed. The assessment was done using the World Health Organization (WHO) hospital assessment tool modified for children's surgery (Children Surgical Assessment Tool, Appendix 1).

The data obtained was analyzed under the 6 WHO health system strengthening pillars of human resource for health, service delivery, infrastructure, information management, finance and leadership and governance. Data analysis was done using the Stata Statistical Software: Release 16. College Station, TX: StataCorp LLC. Ethics approval was obtained from the institutional review board of National Hospital, Abuja, Nigeria.

## Results

The total population of the 4 states included in the evaluation was 41,135,400. Of this population, 43% (17,688,222) were children under the age of 15 years.

### Infrastructure

Children's hospitals are hospitals equipped to specifically and exclusively provide multispecialty medical and surgical care to children and adolescents up till the age of 17 years. Three of the states had no children's hospital. One of the states had a children's hospital but the hospital did not offer any children surgical service. In the entire country, there was no public children's hospital that provides children's surgical services. None of the hospitals had an operating room that was equipped for children's surgery. Of the hospitals evaluated, none had a neonatal intensive care unit (NICU), pediatric intensive care unit (PICU) or a functional ventilator in their intensive care unit. Thirty-one percentage had a special care baby unit and 37.5% had at least one functioning incubator. Intravenous contrast radiology was not available in 71.4% of hospitals, enteral contrast radiology was not available in 67.9% and echocardiography not available in 69.2%. Basic laboratory equipment for complete blood count and serum electrolytes, urea and creatinine were available in all the hospitals.

### Power and water supply

Six (23.1%) out of 26 hospitals evaluated, rarely or never had electric power supply while only 5 (19.2%) always had electric power supply. Of 27 hospitals for which information was available, 5 (18.5%) sometimes or never had running water supply while only 8 (29.6%) always had running water supply.

### Human resources for children's surgery

**Figure 2:**
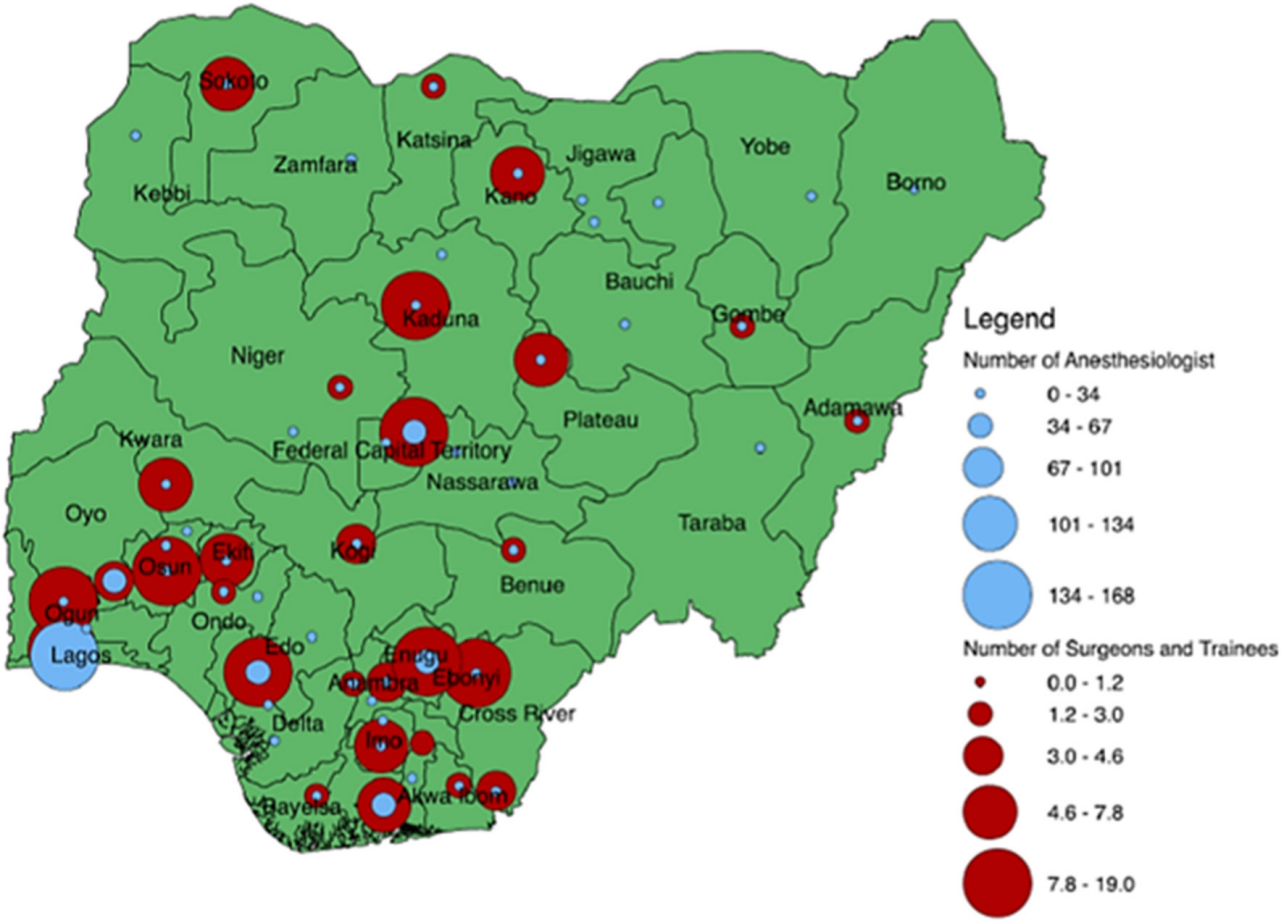
Distribution of paediatric surgeons and anaesthesiologists in Nigeria.

14.3% of the hospitals had a general pediatric surgeon, 14.3% had a general surgeon with pediatric surgery exposure, 71.4% had general duty doctors providing pediatric surgery, 75% had non-physician anaesthesists, 46.4% had general doctors providing pediatric anesthesia and 42.9% had pediatric nurses. In the country, there were 110 pediatric surgeons at the time of this survey and Pediatric surgeon's density was 0.14/100,000 children <15 years. There were 254 registered physician anaesthesiologists, a physician anaesthesiologists density of 0.13/100,000 population and 3 trained pediatric anaesthesiologists. The distribution of pediatric surgeons and anaesthesiologists in the country is summarized in Figure 2.

### Service delivery

Thirty seven percent had adequate 24-h emergency services for children, and only 12% had adequate supply of blood whenever it was needed. Availability of intravenous antibiotics was adequate in 42.3% and none of the hospitals had access to total parenteral nutrition (TPN).

### Information management

Of 28 hospitals, 71.4% used only paper-based record keeping while 28.6% used a combination of both electronic and paper based medical record keeping system.

### Finance

Overall country information indicated that <5% of the population are enrolled in the national health insurance scheme (7). Out of pocket expenditure and catastrophic health expenditure could not be evaluated. In 50% of the hospitals, none of their patients had health insurance while in another 42%, <25% had health insurance. The median cost of herniotomy was N15000 ($41.55) (IQR N10000–N20000) (IQR $27.70–$55.40). The median cost of pediatric colostomy was N25000 ($69.25) (IQR N15000–N86500) (IQR $41.55–$239.61). During this period, the minimum wage in the country was N30000 ($83.10).

### Leadership and governance

Of 26 hospitals evaluated, 8 (46.2%) rarely or never collect data on surgical complications, while only 19.3% always collect data on their surgical complications. Twenty-five hospitals provided information on peri-operative mortality. Ten hospitals (40%) never or rarely collect data on peri-operative mortality while only 6 (24%) always collect data on perioperative mortality. Of 26 hospitals that had information on morbidity and mortality meetings, 6 (23.1%) never have a morbidity and mortality review meeting, 1 (3.9%) have such meetings annually, 1 (3.9%) quarterly, 14 (54%) monthly, 2 (7.7%) every 2 weeks and 2 (7.7%) weekly. Among 24 hospitals, 11 (46.0%) never or rarely use the WHO surgical safety checklist while only 3 (12.5%) always use the checklist.

## Incorporating children's surgery into the national surgical, obstetrics, anesthesia and nursing plan

The following key gaps were identified from the baseline assessment:

Infrastructure gap: lack of children's hospitals providing surgical care, lack of appropriate equipment for children's surgical care.

Human resource for children's surgery: low density of pediatric surgeons and anaesthesiologists.

Service delivery: poor emergency preparedness.

Health information management: lack of reliable data.

Leadership and governance: Non-inclusion of children's surgery in national health plan.

Based on these gaps, strategic goals and priorities were created for children's surgery including

1. Scaling up infrastructure (children's hospital, children's friendly environment).
2. Scaling up human resources for children's surgical care (training general surgeons and non-specialist physicians, pediatric surgeons, pediatric anaesthesiologists).
3. Creation of essential children surgical package at district hospital level.
4. Creation of efficient children's pre-hospital care.
5. Strengthening critical care for children.
6. Development of reliable data and surveillance system and scaling up research capacity.

### Including children surgery in the NSOANP

Using the Optimal Resources for Children's Surgical Care (OReCS) document (8) and focusing on the strategic goals and priorities, children's surgery was incorporated into the NSOANP.

### Pilot implementation

After creation and launching of the NSOANP document, a pilot implementation was undertaken. To facilitate this, key entry points were developed based on the priorities. These entry points included infrastructure, training, critical care, data and research capacity. Outcome of the pilot implementation include the following

1. Creation of children's operating rooms in 4 tertiary hospitals. This has shortened backlogs of elective surgeries and increased the availability of high-quality children specific equipment and instruments with a resultant improvement in the motivation of the surgical team.
2. Creation of a curriculum for training of non-specialist physicians to provide emergency surgical care for children. The curriculum has been used to generate a training manual and a training of trainers programme for specialists has been instituted for training the non-specialist physicians.
3. Training of 92 doctors in basic and pediatric advanced life support.
4. Training of nurses in safe perioperative nursing (24 had physical hands-on training while 906 had virtual training).
5. Creation and deployment of an electronic registry to capture surgical data at hospital level. This has started with collection of data on cleft lip and palate care. The registry will be gradually scaled up over the next several years to capture other birth defects.
6. Training of 54 surgeons, anaesthestists, nurses and other support staff in the fundamentals of research, grants writing and publication.

The impact of above pilot implementation will be evaluated as part of review and revision of the current surgical plan. The evaluation will be done at both facility and country level.

## Discussion

### Lack of inclusion and prioritization of children's surgery in health plans

It has been shown that most LMIC country health plans as well as NSOAPs that have been created do not explicitly prioritize children's surgical care (9). This was also the finding in Nigeria despite children and adolescents constituting up to nearly 50% of the population in this setting (5). Without prioritizing children's surgery within health plans, it would be difficult for governments and development partners to invest in the provision of surgical care for children (10).

### Lessons from Nigeria

#### Gaps

There are several important gaps in children surgical care in Nigeria were identified. The lack of functional public children's hospitals that provides surgical care is worrisome as this would limit progress in training, quality improvement and research (11). This is further compounded by near total lack of children specific equipment and instruments as well as consumables and supplies at the district hospital level. It has been previously reported that many common emergency and essential surgical procedures can be delivered at the district hospital level (1). The lack of appropriate infrastructure and supplies imply that this cannot be realized with the current state of district hospitals in Nigeria.

The density of pediatric surgeons and physician anaesthesiologists is far below what is required to address the large burden of children's surgical conditions in this setting (12). The recommended pediatric surgeon's density is 1 per 100,000 population below 15 years (12). The density from the baseline assessment was 0.14 per 100,000 population <15 years while density of anaesthesiologists was 0.13 per 100,000 population (9). This is further compounded by maldistribution of the few available paeditric surgeons and physician anaesthesiologists leaving large parts of the country without access (13).

At the moment, there is no clear-cut package of children surgical procedures to be delivered at district hospital level as there has been no agreement on what should constitute bellwether procedures for children unlike in adults (14). This would make planning training and allocation of resources at that level difficult.

#### Advocacy

To influence policy on children's surgery, it is crucial that children surgery providers are involved in health and surgical system strengthening programmes. In Nigeria, pediatric surgeons played key leadership roles in the process of developing the NSOANP. This enabled specific prioritization and inclusion of children's surgery. International voices and collaborations are also important to support local efforts., The Global Initiative for Children's Surgery (GICS), an organization at the forefront of advocacy for children's surgery, was helpful in supporting inclusion of children's surgery using the OReCS document.

#### Role of pilot implementation

An important concern about NSOAPs has been the high cost of implementation which has hampered the process. To demonstrate the feasibility of implementation, Nigeria deployed a pilot implementation of the plan through a few key entry points. The experience not only demonstrated that implementation is both feasible and achievable, but that plans need to be broken down into realistic and manageable implementation components that will guide funding drive and allocation of resources by government. This has made it possible for Nigeria to move from pilot implementation to actual implementation of different components of the plan. This experience will be useful for other countries that have developed NSOAPs or are creating NSOAPs as well as encourage governments and development partners to invest in strengthening surgical care for children within their existing health plans.

#### Sustainability

In LMICs, sustaining the implementation of initiatives, innovations and policies has always been a challenge. To ensure that children's surgical programmes are sustained, ongoing advocacy and engagement with public child health communities, policy makers as well as funding and development partners needs to be prioritized and sustained. Local communities should also be part of the advocacy drive to strengthen children's surgical care. These efforts will ensure that children's surgery providers move beyond the current cycle of “speaking to ourselves” to truly global engagement.

## Conclusion

Experience from Nigeria has shown that with appropriate advocacy and the involvement of children surgery providers, children's surgery can be prioritized and implemented within country surgical and health plans. Support from global child health organizations is crucial in achieving this.

## Data availability statement

The raw data supporting the conclusions of this article will be made available by the authors, without undue reservation.

## Author contributions

All authors contributed to the conceptualization, drafting and critical review of the manuscript and approved the final version. All authors are accountable for all aspects of the work.
