# Supplementary material for: Advocating for children's surgery within country health plans: lessons from Nigeria and the global stage
Source: Front Public Health. 2023 Aug 8;11:1209902. doi: 10.3389/fpubh.2023.1209902 (PMC10442532; doi:10.3389/fpubh.2023.1209902)
Supplement: Supplementary file 1 [file Data_Sheet_1.PDF]

WHO-PGSSC-GICS CHILDREN'S SURGICAL ASSESSMENT TOOL (CSAT)

FACILITY CHARACTERISTICS (for patients <15 years old)	
Total number of children's surgical admissions in a year	#
Total number of children's surgical outpatients (visits) seen in a year	#
Total number of hospital beds dedicated to children's surgery	#
Total number children's operating rooms	#
Total number of neonatal unit/special baby care unit beds	#
Total number of paediatric ICU/advanced care beds	#
Total number of neonatal ICU beds	#
Total number of functional paediatric ventilators in the ICU	#
Total number of functional neonatal ventilators in ICU	#
Total number of functional incubators	#
Total number of patient monitors in paediatric and neonatal ICU	#
Access and referral systems:	
How many patients per year do you refer to higher-level facilities for surgical interventions?	#
What is the most common reason for referral of paediatric surgical patients to higher levels of care?	

INFRASTRUCTURE

General Infrastructure - **How often is this item available <u>and</u> functional? Choose (tick)**<br>0- Unavailable (**NOT AVAILABLE** under any circumstances);<br>1- Inadequate (available to **LESS THAN HALF** of the time);<br>2- Limited (available to **MORE THAN HALF**, of the time but not all of the time); or<br>3- Adequate (**AVAILABLE** all of the time without restrictions).	Unavailable (0)	Inadequate (1)	Limited (2)	Adequate (3)
24-hour Emergency Unit able to receive paediatric patients	0	1	2	3
Pharmacy **–** product availability				
Parenteral nutrition	0	1	2	3
Intravenous antibiotics				
Radiology & Pathology – service availability				
Enteral contrast material for bowel evaluation (barium or gastrografin)	0	1	2	3
Intravenous contrast material				
Echocardiogram	0	1	2	3
Anatomic Pathology services	0	1	2	3
Blood Supply - availability				
Blood component transfusion	0	1	2	3

SERVICE DELIVERY

Rate adequacy as below:

0- Unavailable (**NOT AVAILABLE** under any circumstances);
1- Inadequate (available to **LESS THAN HALF** of the time);
2- Limited (available to **MORE THAN HALF**, of the time but not all of the time)
3- Adequate (**AVAILABLE** all of the time without restrictions).

If Unavailable, Inadequate or Limited, please identify the barriers to access (check all that apply)
Infrastructure - physical space, equipment or materials; **Absent** - has never has been present; **Broken** –resources present, but broken; **Personnel** - resource, service or function available, and staff trained, but limited availability at times (eg, night, weekend or holiday); **Training** - No staff trained in using resource or performing function; **Stock out** - cannot be procured, or required equipment or supplies out of stock often due to poor stock management practices or procurement failures; **User fees** - available, but out-of-pocket payment requirement prevents delivery for some; **Other** - Other factors

	Total # performed / year	Rate (0-3)	**Infrastructure**	**Absent**	**Broken**	**Personnel**	**Training**	**Stock out**	**User fees**	**Other**
**Procedures- Minor in patients <15 years old**										
Suturing laceration	#									
Drainage of superficial abscess	#									
Wound debridement	#									
Biopsy (tumour)	#									
Male circumcision	#									
Management of non-displaced fractures	#									
Removal of foreign body from ear/nose	#									
Removal of foreign body from throat										
Eyelid surgery for trachoma	#									

**Procedures – Major in patients < 15 years old**										
Children's surgery										
Appendectomy	#									
Hernia/ hydrocele repair	#									
Reduction of intussusception	#									
Bowel resection	#									
Gastroschisis & omphalocele	#									
Rectal biopsy	#									
Resection of solid abdominal masses	#									
Creation of Intestinal stomas	#									
Closure of intestinal stomas	#									
Pyloromyotomy	#									
Catheterization/ suprapubic cystostomy	#									
Orchiopexy	#									
Repair of testicular or ovarian torsion	#									
Thyroidectomy *(total or partial)*	#									
Drainage of septic arthritis/osteomyelitis	#									
Repair of cleft lip and/or palate	#									
Paediatric Resuscitation & Injury										
Central lines	#									
Surgical airway (cricothyroidotomy, tracheostomy)	#									
Tube thoracostomy	#									
Trauma laparotomy	#									
Open reduction & internal fixation	#									
Placement of paediatric external fixator	#									
Escharotomy/fasciotomy/ contracture release	#									
Amputations	#									
Skin grafting	#									
Burr hole	#									
Craniotomy	#									

**Procedures - Advanced (<15 years old)**										
Repair of esophageal atresia	#									
Repair of intestinal atresia	#									
Myelomeningocele repair	#									
Repair of club foot	#									
Atrial / Ventricular Septal Defect repair	#									
VP Shunt/ETV for hydrocephalus	#									

Repair of anorectal malformation or Hirschsprung's Disease	#									

Surgical Volume

Number of paediatric laparotomies (<15 y/o) performed last year	#
Number of neonatal (< 1 month age) stomas performed last year	#
Number of surgical repairs of paediatric fractures performed last year	#
Total number of procedures performed in pediatric patients (<15 years) last year	#
Percent of children's surgery cases that were emergent or urgent (non-elective) cases	%

Quality and Safety

Is your institution involved in a formal training program for surgical trainees?	☐ Yes ☐ No
Does your institution have a method of monitoring surgical outcomes over time?	☐ Yes ☐ No
Does your institution use electronic medical records?	☐ Yes ☐ No
Does your institution have a trauma registry that includes paediatric trauma?	☐ Yes ☐ No
Number of post-operative paediatric (<15 y/o) in-hospital deaths last year	#
Number of surgical site infections(SSIs) in paediatric patients (< 15 y/o) in the last year	#
What is the highest ASA class of children operated at your institution?	☐ 1 ☐ 2 ☐ 3 ☐ 4
Is the WHO Surgical Safety Checklist used in the operating rooms for paediatric patients?	☐ Never ☐ Less than half the time<br>☐ More than half the time ☐ All the time
Is pulse oximetry used in the operating rooms for paediatric patients?	☐ Never ☐ Less than half the time<br>☐ More than half the time ☐ All the time
How often does the hospital hold a surgical audit (Mortality and Morbidity conference) related to children's surgical patients?	☐ Never ☐ Every week<br>☐ Every month ☐ Every quarter<br>☐ As needed ☐ Other<br>________________

Operating Room Equipment and Supplies – How often is the following equipment available and functional for surgery?

	Rating (0-3)	**Infra-structure**	**Absent**	**Broken**	**Person-nel**	**Training**	**Stock out**	**User fees**	**Other**
Functional paediatric anaesthesia machines									
Paediatric oropharyngeal airway *(000-4)*									
Paediatric endotracheal tubes *(2.5 - 6 mm)*									
Paediatric laryngoscope *(Miller ≤ 2 or Macintosh ≤ 3)*									
Paediatric facemask bag valve *(< 550ml bag with < size 3 mask)*									
Paediatric difficult airway kit (LMA)									
Paediatric Magill forceps									
Paediatric blood pressure monitor or cuff									
Paediatric pulse oximetry									
Paediatric nasogastric Tube *(<12 Fr)*									
Paediatric chest tubes *(< 20 Fr)*									
Paediatric surgical instruments									
Paediatric urinary catheters *(<12 Fr)*									
Paediatric central lines *(< 7 Fr or 12 cm)*									
Sutures *(4.0, 5.0, 6.0)*									
Minimally invasive surgical instruments *(ie. laparoscopy, thoracoscopy, arthroscopy)*									
Capnography (exp. CO_2 measurement)									
Pre-formed intestinal silos									

WORKFORCE

Children's Surgery Provider Density

Providers	Full-time	Part-time
Number of qualified general surgeons with paediatric exposure (*trained surgeons with expertise in pediatric or neonatal surgery, but without formal subspecialty pediatric surgical training)*	#	#
Number of qualified general paediatric surgeons (*trained surgeons with formal specialized training in children's surgery of ≥ 1 year)*	#	#
Number of general doctors providing paediatric surgery (*general practitioners without formal surgical training)*	#	#
Number of non-physicians providing paediatric surgery (*non-physicians health care professional who performs surgery independently without formal training in surgery)*	#	#
Number of qualified paediatric anaesthesiologists (*trained anaesthesiologists with formal specialization in pediatric anesthesia of ≥1 year)*	#	#
Number of qualified anaesthesiologists with paediatric exposure (*trained anaesthesiologists with experience in pediatric anesthesia without formal subspecialty pediatric anaesthesiology training.)*	#	#
Number of general doctors providing paediatric anaesthesia (*general practitioners without formal anaesthesiology training)*	#	#
Number of non-physicians providing paediatric anaesthesia (*non-physicians health care professionals who perform paediatric anaesthesia independently without formal training in anaesthesia)*	#	#
Number of nurses treating only children	#	#

Please identify paediatric specialists that are available at your hospital *(indicate number in brackets)*

- ☐ cardiac surgeon (   )
- ☐ dental surgeon (   )
- ☐ neurosurgeon (   )
- ☐ ophthalmologist (   )
- ☐ orthopedic surgeon (   )
- ☐ otorhinolaryngologist (ENT) (   )
- ☐ plastic surgeon (   )
- ☐ urologist (   )
- ☐ general paediatrician (   )
- ☐ neonatologist (   )
- ☐ cardiologist (   )
- ☐ endocrinologist (   )
- ☐ haematologist (   )
- ☐ nephrologist (   )
- ☐ neurologist (   )
- ☐ respiratory physician (   )

Please identify staff members that are present in your hospital *(indicate number in brackets)*

- ☐ paediatric intensive care nurse (   )
- ☐ neonatal nurse (   )
- ☐ operating room nurse (   )
- ☐ qualified nutritionist (   )
- ☐ radiographer (   )
- ☐ speech therapist (   )
- ☐ audiometrist (   )
- ☐ radiologist (   )
- ☐ pathologist (   )
- ☐ oncologist treating children (   )

**Work Force Availability** (*How often are these available 24 hours a day?)*	Unavailable (0)	Inadequate (1)	Limite d (2)	Adequate (3)
General children's surgical provider availability				
Paediatric anaesthesia provider availability				

FINANCING

Health financing and accounting

What percentage of children coming to this hospital have health insurance?	☐ None ☐ Less than half ☐ More than half ☐ All the time
Nationally, is there government-sponsored health insurance/financing for children?	☐ Yes ☐ No

Budget Allocation

Annual hospital budget allotted to children's surgery and anaesthesia ☐ N/A	Actual amount: % of total budget:

**Cost**: average total inpatient cost for a patient for…	**Cost**	**% out of pocket**
paediatric hernia repair		
paediatric open fracture repair		
paediatric laparotomy		
repair of Hirschsprung's disease/anorectal malformation		

TRAINING AND RESEARCH

How many ongoing research projects involve children's surgery?	#
How many ongoing research projects involve paediatric anaesthesia?	#
How many ongoing research projects involve paediatric nursing?	#
How many workshops, trainings, and lectures are in an average month?	#
